# Mental Health Among Spanish Doctoral Students: Relationship Between Anxiety, Depression, Life Satisfaction, and Mentoring

**DOI:** 10.3390/ejihpe15080164

**Published:** 2025-08-17

**Authors:** Virginia Krieger, Cristina Cañete-Massé, Juan Antonio Amador-Campos, Maribel Peró-Cebollero, María Feliu-Torruella, Alba Pérez-González, Adolfo José Jarne-Esparcia, Xavier María Triadó-Ivern, Joan Guàrdia-Olmos

**Affiliations:** 1 Department of Clinical Psychology and Psychobiology, Faculty of Psychology, University of Barcelona, 08035 Barcelona, Spain; v.krieger@ub.edu (V.K.); ajarne@ub.edu (A.J.J.-E.); 2 Department of Social Psychology and Quantitative Psychology, University of Barcelona, 08035 Barcelona, Spain; cristinacanete@ub.edu (C.C.-M.); mpero@ub.edu (M.P.-C.); jguardia@ub.edu (J.G.-O.); 3 Institute of Neuroscience, University of Barcelona (UBneuro), 08035 Barcelona, Spain; 4 Universitat de Barcelona Institute of Complex Systems (UBICS), Universitat de Barcelona, 08035 Barcelona, Spain; 5 Department of Applied Didactics, Faculty of Education, University of Barcelona, 08035 Barcelona, Spain; mfeliu@ub.edu; 6 Faculty of Psychology and Educational Sciences, Open University of Catalonia, 08035 Barcelona, Spain; albaperezgonzalez@uoc.edu; 7 Business Department, Faculty of Economics and Business, University of Barcelona, 08035 Barcelona, Spain; xtriado@ub.edu

**Keywords:** doctoral students, anxiety, depression, life satisfaction, mentoring

## Abstract

Background: Mental health issues among PhD students are rising, a trend believed to be driven by academic and social challenges. Method: A total of 1265 doctorate students from a large university in Barcelona, Spain (739 women; 414 men; 112 marked other options), with a mean age of 32.36 years (SD = 8.20, range: 23–67), were evaluated by means of standardized instruments. Results: Totals of 40.6% and 46.5% of the sample exceeded the cut-off point for anxiety and depression symptoms, and 57.7% for life satisfaction. The proportion of females exceeding the cut-off point was significantly higher than that of males for both anxiety (women: 43.8%, men: 34.5%) and depression (women: 49.3%, men: 39.8%), but not for life satisfaction (women: 57.6%, men: 58.4%). Arts and Humanities PhD students’ disciplines reported higher anxiety and depression scores than those in Social Sciences, Experimental Sciences and Mathematics, and Health Sciences, respectively, while Social Sciences students showed higher life satisfaction and mentoring support than the other groups. Depression scores were significant predictors of life satisfaction across all doctoral programs. Conclusions: These findings highlight the importance of mentoring in supporting doctoral students’ mental health and life satisfaction and can also inform policies in educational institutions, given that PhD students experiencing psychopathological disorders are at a higher risk of academic failure and dropout.

## 1. Introduction

There is an increased risk of mental disorders during early adulthood, and many psychiatric conditions have an onset at 17–24 years of age (Kessler et al., 2007). The university period appears to be a critical time for the emergence of mental health challenges among young people (Long et al., 2021). Over the last decade, studies have found that 19.2–32% of university students reported mental health issues and subsyndromal symptoms of psychological disorders (Auerbach et al., 2018; Karyotaki et al., 2020). Indeed, research has indicated that university students experience high levels of depression and anxiety (Ahmed et al., 2023; Beiter et al., 2015), low life satisfaction (Paschali & Tsitsas, 2010), exposure to traumatic events (Davies et al., 2022; Ibrahim et al., 2013), and an increased risk of suicidal behaviors (Gulec Oyekcin et al., 2017; Rotenstein et al., 2016).

In this context, doctoral students are widely acknowledged as a high-risk group for mental health issues within the university community, as they face unique challenges such as training periods of unpredictable duration, financial strain, and a lack of a sense of belonging (Barry et al., 2018; Berry et al., 2022; X. K. Liu et al., 2020; Richardson et al., 2018). In addition to these challenges, many doctoral students must also juggle their academic responsibilities with other significant roles, such as being employees, parents, or caregivers (C. Liu et al., 2019; Martinez et al., 2013; Schmidt & Umans, 2014). Recent research addressing mental health issues among PhD students has consistently reported elevated levels of psychological distress, along with symptoms of anxiety and depression, suicide-related outcomes, and reduced life satisfaction (Hazell et al., 2020; Friedrich et al., 2023; Heming et al., 2024; Keloharju et al., 2024; Mahsood et al., 2025; Satinsky et al., 2021; Sverdlik et al., 2018). In this regard, Levecque et al. (2017) reported that 51% of PhD students exhibited at least two symptoms of depression, 40% reported at least three symptoms, and 32% experienced at least four symptoms. The prevalence of having or developing a common psychiatric disorder (i.e., depression) was 2.43 times higher in PhD students compared with highly educated participants in the general population, 2.84 times higher compared with highly educated employees, and 1.85 times higher compared with higher education students.

A large and growing body of evidence suggests that mental health problems among doctoral students are observed across a wide range of PhD subject areas (Devos et al., 2017; Evans et al., 2018; Levecque et al., 2017; Nagy et al., 2019; Satinsky et al., 2021). Specifically, the literature points to high rates of depression and anxiety symptoms as well as psychological distress among students enrolled in STEM disciplines (Science, Technology, Engineering, and Mathematics), business and finance, and humanities and Social Sciences (Devos et al., 2017; Evans et al., 2018; Hazell et al., 2020; Levecque et al., 2017; Nagy et al., 2019). Findings on whether mental health problems differ according to PhD subject area are mixed. For instance, Nagy et al. (2019) examined mental health issues among biomedical doctoral students (*n* = 69) and found that they had a lower prevalence of moderate to severe depressive symptoms (10.1%) compared with their peers in economics. Moreover, 47.6% of biomedical students with anxiety disorders reported having used mental health services in the past year, significantly more than the 21% of economics students reported using these services. In contrast, Levecque et al. (2017) found no significant differences in depression symptoms or psychological distress across different academic disciplines among doctoral students.

To date, studies addressing the relationship between symptoms of anxiety and depression and life satisfaction among doctoral students remain scarce (e.g., Friedrich et al., 2023; Mahsood et al., 2025; Ooi et al., 2022). However, existing research has shown that depression and anxiety symptoms negatively predict or have an adverse effect on life satisfaction among university students (X. Liu & Wang, 2024; Ooi et al., 2022). This point is particularly relevant, as life satisfaction, conceptualized as a broad cognitive appraisal of the overall quality of one’s life, appears to be a major component of subjective well-being (Pavot & Diener, 2008). In the context of higher education, doctoral satisfaction refers to a student’s subjective perception of whether their doctoral experience meets their personal expectations (Teng et al., 2025). Difficulties in life satisfaction may be related to mental health issues and can have detrimental effects on adaptation to university demands and satisfaction with university life (e.g., X. Liu & Wang, 2024).

It has been acknowledged that female doctoral students are at greater risk of experiencing elevated levels of stress, emotional irritability, and symptoms of anxiety and depression compared with their male counterparts (Evans et al., 2018; Friedrich et al., 2023; Müller et al., 2022; Satinsky et al., 2021). In this sense, Hazell et al. (2021), using a mixed-methods approach to examine the mental health of PhD students, found that women face a significantly higher risk of experiencing a range of mental health challenges, both clinical and subclinical, compared with the general population. Furthermore, the research outcomes suggest that female PhD students, in comparison to males, report lower overall satisfaction, likely because of a mismatch between gendered expectations, academic demands, and work–life balance, among other challenges encountered during their doctoral studies (Teng et al., 2025). Most previous studies examining the impact of PhD mentoring experiences on doctoral students’ mental health problems show that the quality of the mentoring relationship or supervisor’s leadership style is associated with depression and anxiety symptoms (Friedrich et al., 2023; C. Liu et al., 2019). Additionally, the advisor–student relationship has been found to partially mediate the association between stress and burnout (Hish et al., 2019), and effective mentorship has been linked to improved mental well-being and greater life satisfaction among doctoral students (Al Makhamreh & Stockley, 2020). In this sense, Nature’s survey of more than 5700 doctoral students highlights that mentoring, particularly in the form of guidance and recognition, contributed more to respondents’ overall satisfaction with their doctoral programs than any other factor (Woolston, 2017).

Research on the mental health of doctoral student populations has predominantly focused on countries such as the United States, the United Kingdom, Finland, and Australia (Hazell et al., 2020; Satinsky et al., 2021). In contrast, there is a notable lack of research in countries such as Spain, highlighting a significant gap in population-based studies on this topic. In line with the above, Estupiñá et al. (2024) found that between 50% and 60% of Spanish university students enrolled in programs such as Health Sciences (23.77%), Arts and Humanities (27.31%), Sciences (19.94%), Social and Legal Sciences (23.38%), and Engineering and Architecture (3.44%) may be experiencing a common psychological disorder. Additionally, 43.6% of participants reported symptoms of depression, while 58.7% reported symptoms of anxiety. This study also found that being female and having low life satisfaction are significant predictors of mental health problems among PhD students, whereas satisfaction with the supervisor is not a significant predictor. Prieto-Vila et al. (2024) reported gender differences between female and male PhD students, with female candidates exhibiting poorer mental health, primarily characterized by psychological distress and symptoms of anxiety. For their part, Sorrel et al. (2020) found that, in addition to 35.8% of students reporting symptoms of anxiety and depression, the majority also exhibited high levels of emotional exhaustion (80.3%) and reduced personal accomplishment (58.9%). The same study also highlights negative relationships between academic advising (supervisor support) and some variables related to mental health issues. Finally, Jiménez-Villamizar et al. (2025) reported elevated symptoms of emotional distress, including depression, negative affect, anger, and anxiety, alongside lower life satisfaction among Spanish PhD students compared with their Mexican counterparts.

Considering the above and given that data on the Spanish population remain scarce, this study aims to build on existing findings and address the gap in the previously studied research population. Therefore, one of the objectives of this study was to examine the prevalence of depressive and anxiety symptoms, as well as overall life satisfaction, among doctoral students from different PhD subject areas at a major university in Barcelona, Spain. Additionally, we hypothesized that: (1) doctoral students in Health Sciences, and Experimental Sciences and Mathematics would score higher on measures of anxiety and depression and lower on measures of life satisfaction and mentoring quality, compared with those in Social Sciences and Arts and Humanities; and (2) gender, along with depressive and anxiety symptoms and the quality of mentoring would significantly predict life satisfaction across PhD subject areas.

## 2. Materials and Methods

### 2.1. Participants

The sample comprised 1265 doctoral students (739 women, 414 men, 5 non-binary, 4 identifying as ‘other’, 22 who preferred not to respond, and 81 who did not answer), with a mean age of 32.36 years (SD = 8.20; range: 23–67). All participants were enrolled in one of 46 doctoral programs across Health Sciences, Experimental Sciences and Mathematics, Social Sciences, and Arts and Humanities at the University of Barcelona (Spain), and completed a survey on emotional well-being, academic performance, and thesis supervision. The population of doctoral students at this university is 5007; thus, 25.26% of PhD students responded.

Our inclusion criteria required that participants were still enrolled in a doctoral program, had not yet earned a doctoral degree, and had completed all or part of the online survey measures.

### 2.2. Instruments

Generalized Anxiety Disorder-7, GAD-7 (García-Campayo et al., 2010; Spitzer et al., 2006). The GAD-7 is a self-report questionnaire used to screen the presence and frequency of anxiety symptoms in the past 2 weeks of one’s daily life. It contains seven items, which are scored from 0 (not at all) to 3 (almost every day). The total score on the GAD-7 is the sum of the item scores (range zero to 21). A higher score indicates more anxiety symptoms, with a score ≥ 10 indicating the threshold for GAD. This cut-off point is used because it has been shown to have high sensitivity (89%) and specificity (82%) (Spitzer et al., 2006). The internal consistency of the GAD-7 (García-Campayo et al., 2010) has previously been reported as excellent (Cronbach α = 0.94). In the current study, Cronbach’s α was excellent, at 0.91.

Center for Epidemiologic Studies Depression Scale—Revised, CESD-R (Van Dam & Earleywine, 2011). The CESD-R consists of 20 items that closely reflect the DSM-IV and DSM-5 diagnostic criteria for depression (APA, 2013). Items have five response options: Not at all or less than 1 day during last week (0), 1–2 days during last week (1), 3–4 days during last week (2), 5–7 days during last week (3), and almost every day for 2 weeks (4). The total score on the CESD-R is the sum of the item scores. The range of possible scores is 0 to 80. A higher score indicates the presence of more depressive symptoms. A total score < 16 indicates no clinical significance, while ≥16 indicates the subthreshold for depression symptoms (Eaton & Kessler, 1981). The CESD-R has good psychometric properties: internal consistency has previously been reported to be high (Cronbach α = 0.93). In the current study, the reliability was excellent (Cronbach α = 0.93).

Satisfaction With Life Scale, SWLS (Diener et al., 1985; Moyano-Díaz et al., 2014). The SWLS is a self-report questionnaire designed to measure global life satisfaction. It consists of five items; each rated on a 7-point Likert scale (1 = strongly disagree to 7 = strongly agree). The total score ranges from 5 to 35, with higher scores indicating greater life satisfaction. Score interpretations are as follows: 31–35 = extremely satisfied, 26–30 = satisfied, 21–25 = slightly satisfied, 20 = neutral, 15–19 = slightly dissatisfied, 10–14 = dissatisfied, and 5–9 = extremely dissatisfied (Pavot & Diener, 1993). The SWLS has demonstrated good internal consistency, with a Cronbach’s α of 0.87 and a test–retest reliability coefficient of 0.82 (Diener et al., 1985; Pavot & Diener, 1993). Studies with samples of Spanish university students have reported acceptable levels of internal consistency, with Cronbach’s α values ranging from 0.81 to 0.84 (Caballero-García & Sánchez Ruiz, 2021). In the current study, the reliability was good (Cronbach α = 0.87)

Mentoring and Thesis Supervision Process Questionnaire, MTSPQ (Amador-Campos et al., 2023). This scale comprises 13 items rated on a 7-point Likert-type scale (1 = strongly disagree; 7 = strongly agree) and assesses doctoral students’ perceptions of the mentoring and supervision they receive from their doctoral thesis supervisor. Higher scores indicate greater satisfaction with the process of directing and supervising a doctoral thesis. In the current study, the reliability was good (Cronbach α = 0.86).

### 2.3. Procedure

Participation in the survey was voluntary, and the sample was obtained through a non-probabilistic convenience sampling method. Data were collected through an online survey administered in a forced-response format using the Qualtrics online survey service provided by the University of Barcelona. At the initial stage of the study, participants received an email containing a comprehensive explanation of the research and an invitation to participate. All students agreed to participate in this study and signed an informed consent before answering the survey by clicking the “I agree” option to consent to participate. The whole process of participation in this study, preparation of the questionnaire, and data processing complied with the General Data Protection Regulation (GDPR) and was endorsed by the Bioethics Committee of the university. The data collection plan involved inviting first-year doctoral students enrolled between September and October 2021 to participate in the online survey. They received the survey link via email on 10 May, followed by a reminder on 15 June 2022. This delay was due to the doctoral school’s policy of granting first-year PhD students an adaptation period lasting until March. Doctoral students in their second to fifth years received the survey link via email on 7 February 2022. A reminder was sent on 21 February 2022, reiterating the purpose of this study and encouraging participation from those who had not yet responded. Access to the online survey was closed on 7 March 2022. Only questionnaires that were fully completed, either for one or all of the measures included in the assessment protocol, were considered valid and included for further analysis. Incomplete questionnaires were excluded. This final criterion led to variations in sample size across the different measures.

This survey was part of an institutional initiative launched in 2018 aimed at assessing and launching, if it were needed, an action plan for improving the mental health and psychological well-being of doctoral students.

### 2.4. Data Analysis

To determine possible relationships between gender and the cut-off points for anxiety and depression symptomology, and life satisfaction, a chi-square test was performed.

A 2 × 4 factorial MANOVA [gender × PhD subject area (Health Sciences, Experimental Sciences and Mathematics, Social Sciences, and Arts and Humanities)] was performed to test whether there were significant differences among the GAD-7, CESD-R, SWLS, and MTSPQ scores according to gender and PhD subject area. Two categories were used for gender (female and male), given the low number of participants who indicated other categories or did not respond. Multivariate analyses were performed using Bonferroni or Games–Howell adjustment for multiple comparisons. Pearson correlations were conducted to explore the relationships between GAD-7, CESD-R, SWLS, and MTSPQ scores, following Cohen’s guidelines for interpreting effect sizes: *r* = 0.10 to 0.29, low; *r* = 0.30 to 0.49, moderate; *r* = 0.50 to 1.0, high (Cohen, 1988). Effect sizes for eta squared (*η*^2^) were interpreted using Cohen’s (1988) criteria: 0.01–0.05, small; 0.06–0.13, medium; ≥0.14, large. Finally, to assess whether gender, symptoms of depression and anxiety, and the quality of the mentoring relationship predicted life satisfaction scores, hierarchical (blockwise entry) regression analyses were performed for each PhD subject area. The adequacy of sample sizes was assessed following common guidelines for subjects per variable (SPV) in linear regression, and all our models met these thresholds, indicating that sample sizes were adequate for the analyses conducted (Austin & Steyerberg, 2015). Regression assumptions were tested and verified, and collinearity diagnostics (Kutner et al., 2005; D. C. Montgomery et al., 2021) were performed to ensure the robustness of the models across the four PhD subject areas. The predictor variables were not highly correlated, and all VIF values were below 5.0, indicating low multicollinearity. The residuals were normally distributed, independent (Durbin–Watson values close to 2), and their variance was constant, suggesting homoscedasticity. Research indicated that women tend to report higher levels of life satisfaction than men (e.g., Joshanloo & Jovanović, 2020; M. Montgomery, 2022). Moreover, consistent with the gender life satisfaction–depression paradox, women are significantly more likely than men to report both greater life satisfaction and higher levels of depressive symptoms (Becchetti & Conzo, 2022). Based on these findings, gender was entered into the model in the first step; in the second step, scores on the anxiety, depression, and mentoring measures were entered.

## 3. Results

Table 1 shows the demographic characteristics of the sample.

Table 2 presents the means and standard deviations of the GAD-7, CESD-R, and SWLS scores, by gender and PhD subject areas. The groups were not equivalent in terms of gender across PhD subject areas [*χ*^2^ (3, 1092) = 36.02; *p* < 0.001]. Specifically, there are more women than men in Health Sciences, Social Sciences, and Art and Humanities.

Prevalence of psychopathological symptoms and life satisfaction

### 3.1. Anxiety

The mean GAD-7 score was 8.79 (*SD* = 5.40; range: 0–21); 40.6% (*n* = 640 of 1022) of the sample exceeded the cut-off point of ≥10. The proportion of females exceeding the cut-off point (43.7%) was significantly higher than that of males (34.5%): *χ*^2^ (1) = 8.161; *p* = 0.004, although the effect size was relatively small (Cramer’s *V* = 0.090).

### 3.2. Depression

The mean CESD-R score was 18.02 (*SD* = 14.85; range: 0–80); 46.5% (*n* = 457 of 980) exceeded the subthreshold cut-off point of 16 for depression (≥16). The proportion of females exceeding the cut-off point (49.3%) was significantly higher than that of males (39.8%): *χ*^2^ (1) = 7.935; *p* = 0.005, although the effect size was relatively small (Cramer’s *V* = 0.091).

### 3.3. Life Satisfaction

The mean SWLS score was 20.85 (*SD* = 6.95; range: 0–35); 57.7% (*n* = 632 of 1095) of the sample exceeded the cut-off point of >20. The proportion of females exceeding the cut-off point (57.6%) was not significantly higher than that of males (58.4%): *χ*^2^ (1) = 0.071; *p* = 0.790, with a low effect size (Cramer’s *V* = 0.008).

### 3.4. Differences in Psychological Symptoms, Life Satisfaction, and Quality of Mentoring Relationship by Gender and PhD Subject Areas

Descriptive statistics for the GAD-7, CESD-R, MTSPQ, and SWLS measures by PhD subject areas are summarized in Table 2.

A MANOVA conducted on the GAD-7, CESD-R, SWLS, and MTSPQ measures revealed no significant interaction between gender and PhD subject areas, Wilks’ λ = 0.979, *F*(12, 2315) = 1.519, *p* = 0.110; *η*2 = 0.007. However, there were significant main effects for gender (Wilks’ λ = 0.984, *F*(4, 875) = 3.815, *p* = 0.004; *η*^2^ = 0.017) and PhD subject areas (Wilks’ λ = 0.929, *F*(12, 2315) = 5.454, *p* < 0.001; *η*^2^ = 0.024).

Univariate analysis revealed significant differences by gender on the GAD-7, *F*(1, 878) = 8.535, *p* = 0.004, *η*^2^ = 0.010, power = 0.831; CESD-R, *F*(1, 878) = 11.707, *p* < 0.001, *η*^2^ = 0.013, power = 0.928. No significant differences were found for the SWLS, *F*(1, 878) = 0.088, *p* = 0.776, *η*^2^ = 0.00, power = 0.060, and the MTSPQ, *F*(1, 878) = 0.00, *p* = 0.989, *η*^2^ = 0.000, power = 0.500. After the Bonferroni post hoc adjustment, women reported higher scores than men on both the GAD-7 and CESD-R measures.

Regarding PhD subject areas, univariate analysis revealed significant differences between groups on all measures: GAD-7, *F*(3, 878) = 4.676, *p* = 0.003, *η*^2^ = 0.016, power = 0.895; CESD-R, *F*(3, 878) = 4.075, *p* = 0.007, *η*^2^ = 0.014, power = 0.846; SWLS, *F*(3, 878 = 5.963, *p* < 0.001, *η*^2^ = 0.020, power = 0.957, and MTSPQ, *F*(3, 878) = 5.830, *p* < 0.001, *η*^2^ = 0.020, power = 0.953. PhD students in Arts and Humanities reported higher scores on the GAD-7 and CESD-R measures, followed by those in Social Sciences, Experimental Sciences and Mathematics, and Health Sciences, respectively. Conversely, students in Social Sciences scored higher on the SWLS and MTSPQ measures compared with their peers in Arts and Humanities, Experimental Sciences and Mathematics, and Health Sciences, respectively.

GAD-7 scores showed high and positive correlations with CESD-R scores, and negative moderate or low correlations with SWLS and MTSPQ scores, respectively. CESD-R scores showed a moderate, negative, and significant correlation with SWLS and a low, negative, but significant correlation with MTSPQ scores. Finally, the correlation between SWLS and MTSPQ scores was moderate and statistically significant (Table 3).

A sequential hierarchical multiple regression analysis was conducted to predict scores on the SWLS scale across the four PhD subject areas: Health Sciences, Experimental Sciences and Mathematics, Social Sciences, and Arts and Humanities. In the first step, gender was entered into the model. In the second step, the GAD-7, CESD-R, and MTSPQ scores were added. The PhD subject area was used as a segmentation variable. Table 4 presents the B, SE B, and β values from the hierarchical regression analysis predicting SWLS scores.

In Health Sciences, SWLS scores were significantly predicted by GAD-7 (β =−0.206, *t* = −3.434, *p* = 0.001, 95% CI [−0.430, −0.117]), CESD-R (β = −0.269, *t* = −4.482, *p* < 0.001, 95% CI [−0.200, −0.078]), and MTSPQ (β = 0.324, *t* = 7.631, *p* < 0.001, 95% CI [0.124, 0.210]), with an *R*^2^ of 0.390, *F*(4, 374) = 59.16, *p* < 0.001. In Experimental Sciences and Mathematics, SWLS scores were predicted by GAD-7 (β = −0.158, *t* = −1.982, *p* = 0.049, 95% CI [−0.383, −0.001]), CESD-R (β = −0.377, *t* = −4.651, *p* < 0.001, 95% CI [−0.239, −0.097]), and MTSPQ (β = 0.230, *t* = 4.032, *p* < 0.001, 95% CI [0.052, 0.151]) with an *R*^2^ of 0.363, *F*(4, 213) = 30.35, *p* < 0.001. For Social Sciences, SWLS scores were predicted only by the CEDS-R (β = −0.455, *t* = −04.634, *p* < 0.001, 95% CI [−0.271, −0.109]) with an *R*^2^ of 0.320, *F*(4, 162) = 18.59, *p* < 0.001. Finally, in Arts and Humanities, SWLS scores were predicted by CESD-R (β = −0.383, *t* = −3.042, *p* = 0.003, 95% CI [−0.277, −0.059] and MTSPQ (β = 0.254, *t* = 3.032, *p* = 0.003, 95% CI [0.041, 0.197]), with an *R*^2^ of 0.212, *F*(4, 117) = 7.88, *p* < 0.001.

## 4. Discussion

Recent evidence suggests that doctoral students often struggle with mental health issues and psychological distress more than the general population, which can significantly diminish their overall well-being and life satisfaction (Friedrich et al., 2023; Hazell et al., 2020; Heming et al., 2024; Mahsood et al., 2025). Additional factors that may influence life satisfaction among PhD students include gender and the nature of the mentoring they receive during their doctoral studies (Estupiñá et al., 2024; Evans et al., 2018; Friedrich et al., 2023). Therefore, this study analyzes, on the one hand, the prevalence of anxiety and depression symptoms, as well as life satisfaction, among doctoral students from different programs and disciplines. On the other hand, it examines the relationships between gender, scores on measures of depressive and anxiety symptoms, life satisfaction, and mentoring quality across different PhD subject areas.

Regarding the prevalence of anxiety and depression symptoms, this study found that 40.6% of participants scored above the clinical cut-off for anxiety, and 46.5% exceeded the threshold for depression. These findings are in line with previous studies analyzing the prevalence of mental health problems among PhD students (Evans et al., 2018; Hazell et al., 2020; Hazell et al., 2021; Levecque et al., 2017) and, importantly, they add to and build upon the work of Sorrel et al. (2020) and Estupiñá et al. (2024), who identified that Spanish doctoral students struggle with high levels of depression and anxiety symptoms. In this regard, the research conducted by the Spanish Ministry of Universities, in collaboration with the Ministry of Health (Ministerio de Universidades, 2023), indicates that 39.4% of Spanish doctoral students report symptoms of depression, while 42.5% report experiencing moderate or severe anxiety. These prevalence rates may reflect the emotional distress and diminished well-being experienced by Spanish doctoral students during the pursuit of their degree, often influenced by challenges such as job uncertainty, academic pressure, and other contributing factors (Jiménez-Villamizar et al., 2025). A recent study on mental health among Mexican and Spanish doctoral students found that Spanish doctoral students reported higher indicators of depression and anxiety symptoms compared with their Mexican counterparts (Jiménez-Villamizar et al., 2025). Other contextual challenges faced by doctoral students include limited professional experience, since many have only recently completed their postgraduate studies (Evans et al., 2018), difficulties in managing socio-family relationships and adapting to work environments (Berry et al., 2021; Evans et al., 2018; Levecque et al., 2017), financial constraints (Ahalli et al., 2022; Berry et al., 2021), and the specific dynamics within departments or research groups (Pyhältö et al., 2012). With regard to life satisfaction, 57.7% of participants scored above the cut-off on the SWLS measure. These findings align with previous studies that have reported good levels of life satisfaction during doctoral studies (Ooi et al., 2022; Xu et al., 2024). However, these findings differ from those reported by Teng et al. (2025), who found lower levels of life satisfaction among female doctoral students compared with their male counterparts, highlighting the intricate challenges that women encounter during the doctoral period.

Furthermore, as expected, our results indicate that women had more symptoms of anxiety and depression than men, which echoes findings from previous studies, both from Spain (Prieto-Vila et al., 2024) and other countries (Evans et al., 2018; Müller et al., 2022). Being female, in combination with other life factors (e.g., social pressure, domestic and/or caring duties, and role conflicts), may represent an increased risk for developing mental health issues (e.g., depression, anxiety, and psychological stress) during the doctoral period compared with male counterparts (Hazell et al., 2020). In this sense, it is important to note that gender differences in anxiety and depression may be partly explained by women’s greater exposure to psychosocial stressors and their potentially higher biological and psychological vulnerability to these conditions (Seedat et al., 2009; Thompson, 2017). Furthermore, in this study, both female and male participants reported high levels of life satisfaction, which contrasts with findings from other studies that have identified gender differences, with females reporting lower satisfaction than males (e.g., Teng et al., 2025). In this context, it is important to note that a study based on data from Nature’s 2019 Global PhD Survey, which included a sample of 6372 students across 108 countries, found that females report lower satisfaction with their doctoral experiences compared with males, with a 3.88% higher likelihood of decreased satisfaction. The same study indicates that this lower satisfaction is associated with factors such as a greater likelihood of experiencing gender discrimination and sexual harassment, overwork, and increased difficulty in balancing work and personal responsibilities (Teng et al., 2025).

Our hypothesis that students in Health Sciences and Experimental Sciences and Mathematics would score higher on anxiety and depression and lower on life satisfaction and mentoring quality, compared with those in Social Sciences and Arts and Humanities, was only partially supported by the results. Unexpectedly, we found that students in Arts and Humanities, followed by those in Social Sciences and Experimental Sciences and Mathematics, reported the highest average scores on anxiety and depression, compared with students in Health Sciences. This may be attributed to the fact that students in Health Sciences report greater access to departmental or government-based financial aid programs compared with their peers in Social Sciences and Arts and Humanities (Sverdlik et al., 2018). Nevertheless, our findings are not aligned with the existing empirical literature that highlights significant mental health challenges—such as symptoms of depression and anxiety, among doctoral students in Biomedical Sciences graduate programs (Lubega et al., 2023; Nagy et al., 2019). Conversely, Levecque et al. (2017), in a representative sample of PhD students (*n* = 3659), found no significant differences in the presentation of psychological distress and depressive symptoms across different doctoral programs, including the Sciences, Biomedical Sciences, Applied Sciences, Humanities, and Social Sciences.

Regarding life satisfaction, students in Social Sciences, followed by those in Arts and Humanities and Health Sciences, reported higher levels of life satisfaction than their peers in Experimental Sciences and Mathematics. These findings contrast with previous studies that reported no significant differences in students’ life satisfaction across various PhD programs (Barnes & Randall, 2012). Finally, students in Social Sciences, followed by those in Arts and Humanities, reported higher levels of mentoring quality on the MTSPQ than students in Health Sciences, as well as those in Experimental Sciences and Mathematics. This is particularly relevant because previous studies examining mentoring relationships in doctoral programs, especially in fields such as Biomedicine, have reported positive and significant associations between high mentoring satisfaction and greater levels of mental well-being (Zhang et al., 2022). As suggested by Lubega et al. (2023), the quality of the mentoring relationship is likely to represent a protective factor for both mental health and academic success among students in STEMM disciplines (Science, Technology, Engineering, Mathematics, and Medicine). In this regard, Evans et al. (2018) emphasize that strong and supportive mentoring relationships between graduate students and their mentors are significantly associated with reduced levels of anxiety and depression. According to Dericks et al. (2019), student satisfaction among doctoral students in the Sciences, Social Sciences, and Humanities is primarily influenced by the department’s academic quality and supportiveness, as well as by the level of support provided by the supervisor.

Finally, we further hypothesized that gender, symptoms of depression and anxiety, and the quality of mentoring would significantly predict life satisfaction across PhD subject areas. These findings partially supported our hypothesis, as the regression analysis indicated that, among the predictors, only CEDS-R scores, compared with the GAD-7 and MTSPQ scores, significantly predicted SWLS scores across the four PhD subject areas. Furthermore, gender did not emerge as a significant predictor of life satisfaction in any of the disciplines. The CEDS-R and GAD-7 scores only predicted SWLS scores in Health Sciences and Experimental Sciences and Mathematics. Furthermore, the MTSPQ scores predicted SWLS scores in Health Sciences, Experimental Sciences and Mathematics, as well as Arts and Humanities. Overall, a heterogeneous and domain-specific pattern of predictions emerged, indicating that symptoms of anxiety and depression, as well as mentoring quality, were stronger predictors of life satisfaction in Health Sciences and Experimental Sciences and Mathematics. These trends suggest that low life satisfaction, one of the most frequently reported indicators of low well-being among doctoral students, may be associated with the doctoral discipline, the quality of mentoring received throughout the doctoral program, and the presence of mental health issues such as internalizing symptoms (e.g., Evans et al., 2018; Friedrich et al., 2023; Lubega et al., 2023; Nagy et al., 2019). It is worth noting that C. Liu et al. (2019) found that mentoring partially mediates the relationship between students’ research self-efficacy and symptoms of depression and anxiety. This finding suggests that mentoring may contribute to improved mental health and research engagement, ultimately enhancing life satisfaction among PhD students (C. Liu et al., 2019; Lunsford, 2012). In this context, Kurtz-Costes et al. (2006) highlight that mentoring often shapes motivational and emotional dimensions of doctoral experiences. Regarding the absence of a predictive relationship between gender and life satisfaction, it may be due to the fact that more than half of the sample, both women and men, reported good life satisfaction. It is possible that this group of PhD students has developed resources to manage and balance the demands of their doctoral studies and personal lives. As a result, they may experience higher life satisfaction.

### 4.1. Limitations

The study design was cross-sectional, providing a snapshot of a process that is longitudinal in nature and may vary over time. Therefore, it did not allow for conclusions regarding causality or the long-term effects of the determinants of life satisfaction among doctoral students. The university’s quality policy has taken this aspect into account and, based on the results of this study, has implemented longitudinal monitoring of the personal well-being and mental health of doctoral students, with the aim of promoting policies that favor the personal well-being of students. In addition, data were collected via self-reports, which may introduce bias and lead to overestimation, potentially undermining accurate evaluation and interpretation of the results. Regarding our sample, although its overall size was relatively large, it represented only 25.3% of the university’s doctoral student population. This, combined with the use of a non-probabilistic convenience sampling method, means that the conclusions drawn are specific to this group and cannot be generalized to a broader population. Future research should employ a probabilistic sampling method to reduce sampling bias. Additionally, the gender imbalance limits the ability to generalize results across genders and may bias the statistical associations observed. As the academic year, which plays a highly significant role in doctoral studies, was not encompassed within this study, future research could explore the heterogeneity of mental health measures across different academic years. In addition, the distribution of PhD subject areas was unbalanced across groups, which warrants caution in interpreting the results. Finally, we only considered two groups in our analyses according to gender (male and female), as the number of people who identified as transgender or gender non-conforming was small.

### 4.2. Conclusions

The present study has shed new light on the prevalence of depression and anxiety symptoms, as well as self-reported life satisfaction, among Spanish PhD students. These findings support the hypothesis that the relationships between mental health issues, mentoring quality, and life satisfaction vary across PhD subject areas. Furthermore, mentoring is a significant predictor of life satisfaction in the PhD subject areas of Health Sciences, Experimental Sciences and Mathematics, and Arts and Humanities. These findings expand on previous research by highlighting the importance of developing university policies that incorporate early assessments of mental health using standardized measures, along with frequent and high-quality mentoring, particularly during the initial stages of doctoral training. Additionally, they emphasize the need for early and targeted interventions to mitigate the impact of these factors on students’ overall life satisfaction.

## Figures and Tables

**Table 1:** Demographic characteristics of the sample.

	*N*	Percentage
Marital status (*n* = 1185)		
Single	552	46.6
Married	225	19.0
Living with a partner	341	28.8
Divorced	23	1.9
Other	44	3.7
Do you have children? (*n* = 1184)		
Yes	192	16.2
No	992	83.8
Caregiving responsibilities (*n* = 1179)		
Yes. Children and/or parents or dependent people	253	21.5
No	926	78.5
Doctoral variables		
Time dedication to PhD (*n* = 1141)		
Full time	852	74.7
Part time	289	25.3
Financing of doctoral studies (*n* = 1140)		
Pre-doctoral contract	668	58.6
Industrial doctorate	4	0.4
Without funding	378	33.1
Other	90	7.9
Do you have a paid occupation related to the thesis? (*n* = 1139)		
Yes	398	34.9
No	741	65.1

Note. Some *n* values do not match due to missing responses for some variables.

**Table 2:** Descriptive statistics of the GAD-7, CESD-R, SWLS, and MTSPQ scores by gender and PhD subject areas (*n* = 1265).

	GAD-7			CESD-R		SWLS		MTSPQ	
PhD Subject Areas	Gender	*n*	Mean (*SD*)	*n*	Mean (*SD*)	*n*	Mean (*SD*)	*n*	Mean (*SD*)
Health Sciences	Female	299	8.18 (5.24)	292	16.60 (13.53)	319	21.08 (6.90)	290	60.61 (13.47)
	Male	11	7.20 (4.57)	118	14.66 (12.46)	129	20.31 (6.94)	112	59.00 (13.71)
Experimental Sciences and Mathematics	Female	115	9.25 (5.62)	110	21.26 (15.99)	120	18.61 (6.45)	114	58.69 (14.81)
	Male	122	7.06 (5.33)	118	16.93 (13.77)	132	20.29 (7.14)	118	61.20 (15.23)
Social Sciences	Female	114	8.17 (5.41)	112	19.02 (15.79)	123	22.95 (6.51)	113	64.30 (13.88)
	Male	73	8.87 (5.84)	65	18.23 (17.07)	76	22.71 (6.96)	69	65.34 (14.24)
Arts and Humanities	Female	98	10.88 (5.24)	90	24.94 (15.69)	102	20.64 (6.66)	93	61.70 (13.80)
	Male	45	8.62 (6.09)	39	18.23 (13.81)	49	21.10 (7.37)	44	65.30 (14.69)

Note. GAD-7 = Generalized Anxiety Disorder-7; CESD-R = Center for Epidemiologic Studies Depression Scale-Revised; SWLS = Satisfaction with Life Scale; MTSPQ = Mentoring and Thesis Supervision Process Questionnaire.

**Table 3:** Correlations between GAD-7, CESD-R, SWLS, and MTSPQ scores.

Measures	1	2	3	4
1. GAD-7	1			
2. CESD-R	0.74 **	1		
3. SWLS	−0.43 *	−0.50 **	1	
4. MTSPQ	−0.18 **	−0.21 **	0.35 **	1

Note. GAD-7 = Generalized Anxiety Disorder-7; CESD-R = Center for Epidemiologic Studies Depression Scale-Revised; SWLS = Satisfaction with Life Scale; MTSPQ = Mentoring and Thesis Supervision Process Questionnaire. * *p* < 0.05, ** *p* < 0.01.

**Table 4:** Summary of significant hierarchical regression analysis for gender, MTSPQ, GAD-7, and CESD-R predicting SWLS scores.

PhD Subject Areas												
	Health Sciences (*n* = 379)			Experimental Sciences and Mathematics (*n* = 218)			Social Sciences (*n* = 167)			Arts and Humanities (*n* = 218)		
	B	SE B	β	B	SE B	β	B	SE B	β	B	SE B	β
SWLS												
STEP 1												
Constant	7.388	1.537		9.082	2.076		19.167	2.761		9.882	3.211	
Gender	−0.360	0.695	−0.024	0.722	0.854	0.054	−0.928	1.076	−0.067	1.457	1.296	0.098
STEP 2												
Constant	16.738	1.804		18.549	2.090		26.868	2.568		16.538	3.519	
Gender	−1.033	0.619	−0.068	−0.208	0.744	−0.016	−0.809	0.914	−0.058	0.041	1.276	0.003
MTSPQ	0.167	0.022	0.324 *	0.102	0.025	0.230 *	0.026	0.032	0.055	0.119	0.039	0.254 *
GAD-7	−0.274	0.080	−0.206 *	−0.192	0.097	−0.158 *	−0.136	0.119	−0.114	0.063	0.151	0.051
CESD-R	−0.139	0.031	−0.269 *	−0.168	0.036	−0.377 *	−0.190	0.041	−0.455 *	−0.168	0.055	−0.383 *

Note. GAD-7 = Generalized Anxiety Disorder-7; CESD-R = Center for Epidemiologic Studies Depression Scale-Revised; SWLS = Satisfaction with Life Scale; MTSPQ = Mentoring and Thesis Supervision Process Questionnaire. * *p* < 0.001.

## Data Availability

Raw data supporting the conclusions of this article will be made available by the authors, without undue reservation.
